# Campbell’s Law Explains the Replication Crisis: Pre-Registration Badges Are History Repeating

**DOI:** 10.1177/10731911241253430

**Published:** 2024-05-23

**Authors:** E. David Klonsky

**Affiliations:** 1 The University of British Columbia, Vancouver, Canada

**Keywords:** pre-registration badges, open science, replication crisis, researcher degrees of freedom, robust science, fragile science, robustness checks

## Abstract

Campbell’s Law explains the replication crisis. In brief, useful tools such as hypotheses, *p*-values, and multi-study designs came to be viewed as indicators of strong science, and thus goals in and of themselves. Consequently, their use became distorted in unanticipated ways (e.g., hypothesizing after results were known [HARKing], p-Hacking, misuses of researcher degrees of freedom), and fragile findings proliferated. Pre-registration mandates are positioned as an antidote. However, I argue that such efforts, perhaps best exemplified by pre-registration badges (PRBs), are history repeating: Another useful tool has been converted into an indicator of strong science and a goal in and of itself. This, too, will distort its use and harm psychological science in unanticipated ways. For example, there is already evidence that papers seeking PRBs routinely violate the rules and spirit of pre-registration. I suggest that pre-registration mandates will (a) discourage optimal scientific practice, (b) exacerbate the file drawer problem, (c) encourage pre-registering after results are known (PRARKing), and (d) create false trust in fragile findings. I conclude that multiple design features can help support replicability (e.g., adequate sample size, valid measurement, robustness checks, pre-registration), none should be canonized, replication is the only arbiter of replicability, and the most important solution is sociocultural: to foster a field that reveres and reinforces robust science—just as we once revered and reinforced flashy but fragile science.

“The first principle [of science] is not to fool yourself—and you

are the easiest person to fool.”

—Richard Feynman

Decades of poor research practices (Simmons et al., 2011) have damaged psychological science and led to a strong desire for change. Perhaps chief among these changes is the development of pre-registration mandates (PRMs). PRMs are policies intended to elevate pre-registration from a tool to a “norm” as an antidote to decades of poor scientific practice (Nosek et al., 2018). PRMs are best exemplified by the pre-registration badge (PRB), which is now awarded by hundreds of journals to papers that pre-register their analytic plan (Center for Open Science [COS], 2023).

In response, this article provides an overarching explanation for the replication crisis and critiques PRMs as a solution. I argue (a) that the field has a long history of converting tools of psychological science into indicators of “strong science” and goals in and of themselves; (b) that this practice is directly responsible for the replication crisis; and (c) that PRMs are the latest iteration of this practice with strong potential to harm rather than safeguard psychological science. I conclude with an alternative vision for the future of psychological science in which replication itself is the arbiter of replicability, various study design features are valued for supporting replicability but none are canonized as badges or goals, and in which robust science is encouraged and reinforced through the same sociocultural processes that have for decades encouraged and reinforced flashy but fragile work.

## The Replication Crisis Is Real

When I argue against PRMs, some may wonder whether I believe the replication crisis is real and profound. I do, absolutely and unequivocally. As I lamented to the Association for Psychological Science (APS, 2014) a decade ago,

Journals, including our field’s top journals, are littered with underpowered studies, *p* < .05 fishing expeditions, and clever or “sexy” conclusions that are not justified by the data—these studies get published as long as they conform to aesthetic norms and yield clever and/or attention-getting headlines . . . Show me five studies in our field’s top psychological science journal, and I’ll show you four with conclusions that can’t be trusted.

This estimate—that only one out of five studies can be trusted—was only slightly too pessimistic. The Open Science Collaboration’s (OSC, 2015) efforts to quantify the replicability of psychological science studies yielded an estimate of 36%. The replication crisis threatens all aspects of psychological science, from basic measurement (Allen et al., 2023; Lilienfeld & Strother, 2020) to applied and clinical psychological science (O’Donohue et al., 2022; Tackett et al., 2019). It must be understood and solved.

## Why Did the Replication Crisis Happen?

So what explains the replication crisis? Is it negligence, in that researchers have accidentally or ignorantly been publishing results unlikely to replicate? Is it fraud, in that researchers have purposely altered data to obtain findings that are false but more likely to be published? Should we blame publication pressure as incentivizing researchers to publish fragile findings? Should we blame journals for prioritizing “significant” results over null or negative results?

All of these and other explanations are relevant. But it can be difficult to make sense of several disparate explanations. If possible, it would be best to have a single, overarching explanation to account for the crisis and inform steps forward. A parsimonious explanation for the replication crisis would inform our choice of solutions—including the potential benefits or unintended negative consequences of PRMs. In this spirit, I offer the following: Campbell’s Law explains the replication crisis.

Campbell’s law suggests that the more an indicator is used for decision-making, the more the indicator will be subjected to corruption pressures, and in turn distort the process it was intended to support (Campbell, 1979). As an illustration, consider the example of standardized educational testing in grade school. Standardized testing was introduced to monitor and safeguard the quality of teaching. However, test results soon became the goal instead of a tool for measuring progress, and there were unforeseen negative consequences. For example, it was observed that teachers came to neglect critical topics such as writing and science in favor of topics emphasized by the standardized tests (e.g., arithmetic, spelling, word recognition) and that teaching methods became those that most mimicked standardized tests rather than those judged to be maximally effective and accessible for students (Smith & Rottenberg, 1991). In short, the goal of providing a good education accidentally morphed into the goal of achieving high test scores. Teaching and education suffered.

How does Campbell’s Law apply to psychological science and the replication crisis? I argue that certain tools designed to support science came to be viewed as indicators of “strong science,” and thus the basis for important decisions such as publication in journals. These indicators then became the new goals, and in turn, the goal of producing strong science morphed into a goal of achieving the indicators. The result was a distortion of optimal scientific practice, decades of fragile science, and an enormous replication crisis.

To illustrate this phenomenon, I will discuss three exemplars: hypotheses, *p*-values, and multi-study designs.

### Hypotheses

Hypotheses have strong, arguably foundational, utility as a tool of science. They support the falsification principle, proposed by Karl Popper as fundamental to the scientific method (Popper, 1963). Hypotheses present testable, refutable predictions that help scientists distinguish more and less accurate ideas.

However, over time, journal editors and reviewers began to value hypothesis-driven research as more desirable than exploratory, descriptive work (even though the latter is also essential to the scientific method). In fact, it was found that journal editors and reviewers came to believe that the large majority of studies submitted to journals should be hypothesis-driven (Kerr, 1998). As a result, hypotheses, once a helpful tool of science, morphed into an indicator of strong, publishable science, and thus a goal in and of themselves. This had a shocking impact on scientific practice.

Psychological scientists began to HARK (hypothesize after results were known; Kerr, 1998). In other words, scientists would complete a study—including data collection, data analysis, and interpretation of findings—and then formulate a hypothesis post hoc to anticipate what they had already found in their data. Exploratory findings were routinely published in the guise of carefully tested hypotheses. Of course, this practice is the opposite in function and spirit to how hypotheses are meant to work in science.

How could valuing a fundamental tool of science harm rather than safeguard the practice of science? A full discussion of the relevant psychological and social forces would be useful but is beyond the scope of this article. However, Campbell’s Law offers an explanation. Once hypotheses were converted from a tool of science into an indicator or symbol of strong science, scientists changed the way they used them. New norms emerged that permitted the use of hypotheses in ways wholly unintended and unanticipated by Popper and like-minded scientists. As a result, HARKing became common and was even encouraged by highly cited scientists (Bem, 2003). At its peak, HARKing may have been more common than genuine hypotheses (Kerr, 1998). In sum, per Campbell’s Law, once hypotheses were elevated from a tool into an indicator, their use became distorted, and their use corrupted (rather than supported) the conduct of psychological science.

### p-Values Below .05

*p*-Values have a similar story. According to the American Statistical Association, *p*-values are a “useful statistical measure” when interpreted appropriately and without overreliance on categorical thresholds (Wasserstein et al., 2016). *p*-Values are easy to compute, easy to report, and thus provide a convenient way to summarize the incompatibility between a particular set of data and a proposed model for the data. For more than 100 years, the *p*-value and its precursors have been used in different ways by different researchers to aid in the interpretation of patterns in data (Kennedy-Shaffer, 2019).

Sometime around the mid-1900s, social scientists started interpreting *p*-values categorically using cutoffs such as <.05 (Kennedy-Shaffer, 2019). This cutoff is widely attributed to Sir Ronald Fisher (Fisher, 1925), who suggested this threshold was “convenient.” However, scientists and journals elevated *p*-values from a tool to an indicator of “strong science.” Study results that achieved *p* < .05 were given preferential treatment: They were referred to as “significant,” often marked by asterisks, and more likely to be published (Masicampo & Lalande, 2012). *p*-Values became a goal instead of a tool. This substantially harmed how researchers conducted science.

In short, psychological scientists used various forms of researcher degrees of freedom, and even fraud, to achieve *p*-values below .05 (Simmons et al., 2011). Phenomena such as *p*-hacking, selective reporting, and publication bias—although all counter to basic ideals of scientific practice—became rampant and littered the scientific literature with false-positive findings (Friese & Frankenbach, 2020; Masicampo & Lalande, 2012). In sum, per Campbell’s law, once *p*-values were elevated from a tool into an indicator of strong science, their use changed and harmed the process they were intended to support.

### Multi-Study Designs

Multi-study designs offer a third potential example of this trend. In general, the inclusion of multiple studies of a given phenomenon in a single paper is viewed as a strength. Multiple studies within the same article can replicate key effects, address each other’s limitations, and examine different potential mechanisms of or explanations for key effects. Thus, a systematic, multi-study design can be an extremely useful scientific tool. However, in some contexts, this approach morphed into an indicator of desirable science and a goal to be achieved, rather than a tool to be used thoughtfully and appropriately.

Consider the *Journal of Personality and Social Psychology* (JPSP) as an exemplar. For 60 years, JPSP has been a premier outlet for empirical studies in social and personality psychology. According to its publisher (American Psychological Association [APA], 2021), JPSP has the most all-time citations in the “Psychology, Social” category and accounts for more than 20% of all citations in this category. JPSP is the juggernaut of social psychology journals.

Why am I highlighting JPSP? Perhaps more than any other psychology journal, JPSP has come to value multiple studies as an essential feature of its publications. In 1982, JPSP articles included a mean of 1.6 studies, but by 2016, the mean was 5.1—and 92% of JPSP articles included multiple studies (Quinones-Vidal et al., 2004; Simon & Wilder, 2022).

One might expect that a 92% rate for multi-study papers would help ensure that published findings are unusually robust. However, in this case, the opposite is true. When the Open Science Collaboration (OSC, 2015) used high-powered designs to attempt replications of dozens of studies from three leading psychology journals, JPSP articles were found to have the lowest replication rate: just 23%.

This result is especially notable because JPSP prioritized not only multi-study papers but also the other two exemplars discussed earlier: hypothesis-driven work yielding *p*-values below .05 (OSC, 2015). In other words, JPSP is a microcosm of the replication crisis. It placed maximal emphasis on three key tools of science—hypotheses, *p*-values, and multi-study designs—and yielded the lowest documented replication rate in psychology. How can this be?

### Summary Explanation for the Replication Crisis

It is my contention that Campbell’s Law offers the most efficient, accurate, and comprehensive explanation for the replication crisis. Once useful tools of science come to be viewed as indicators of strong science, they become goals in and of themselves, and researcher behavior changes to meet those goals in ways that harm scientific practice.

As we look to the future, I believe it is important that we fully benefit from hindsight and embrace the extent to which our behavior as scientists can accidentally be corrupted when the conditions for Campbell’s Law are met. Could Popper have dreamed that hypotheses would come to be formulated after, rather than before, data are analyzed? Could Fisher have imagined that scientists would become so singularly focused on achieving his “convenient”*p*-value threshold that the abuse of researcher degrees of freedom would become common practice? Did JPSP editors imagine that a soft requirement for multi-study investigations could permit the lowest replication rate? We can forgive ourselves and our predecessors for not anticipating these outcomes. But, we should consider ourselves fooled once—and resolve to not be fooled again.

## PRMs Are History Repeating

We now understand what happens when we canonize a scientific tool as an indicator of strong science and a gateway for publication: (a) The tool becomes a goal, (b) Its use becomes distorted, and (c) Scientific practice is harmed. Which brings us to PRMs.

PRMs are extremely well-intended. They have been created to encourage and reward pre-registration, a process by which research questions are defined and an analysis plan created before observing the research outcomes (Nosek et al., 2018). Pre-registration is being framed not as a tool but as a “revolution” in which universal or near-universal use of pre-registration protects against threats to replicability, including the misuse of researcher degrees of freedom, and safeguards against fragile science (Nosek et al., 2018). Hundreds of journals in psychology and allied fields now award badges to articles using pre-registration (OSC, 2023).

However, badges are an explicit example of taking a useful tool, canonizing it as an indicator of strong science, and turning it into a goal. PRMs change pre-registration from a useful tool into a figurative or literal badge to be obtained. This article offers a warning. History and Campbell’s Law tell us how PRMs will impact the field: (a) Pre-registration will become a goal rather than a tool, (b) Its use will be distorted in ways that are unintended and unanticipated, and (c) Scientific practice will be harmed. History is repeating.

To be clear, pre-registration is a wonderful tool—just as hypotheses, *p*-values, and multi-study designs are wonderful tools when used judiciously. Pre-registration can indeed limit researcher degrees of freedom, help investigators think through their analytic plans, and increase transparency for planned versus exploratory analyses. Pre-registration is particularly useful for testing specific hypotheses, including treatment outcome studies and replication studies, although it can be useful for a wide range of both exploratory and confirmatory purposes.

In contrast, as a goal, or a badge, that everyone is encouraged to obtain, PRMs have high potential for harm. Indeed, there is already evidence for the distortion of pre-registration in the service of PRMs. For example, in an early analysis of articles earning PRBs in the journal *Psychological Science*, the vast majority—89%—included undisclosed changes to their pre-registration plan (Claesen et al., 2021). These undisclosed changes were not trivial and involved key researcher degrees of freedom such as the hypothesis being investigated, the variables examined, the exclusion criteria, and the analytic plan. A subsequent analysis of studies given badges or financial incentives for pre-registration focused on hypotheses and found that the majority altered hypotheses between the pre-registered and published versions (van den Akker et al., 2023). There is also a documented case of a journal pro-actively offering a PRB to an article that did not pre-register its key analyses (e.g., Clark et al., 2020, retracted)—perhaps so the journal could achieve its goal of having a high proportion of articles paired with PRBs.

For many, the regularity with which the use of pre-registration is being distorted in service of PRMs may be surprising. One would think those striving to achieve PRMs would be unusually careful and faithful in their implementation of pre-registration. Others may suggest that these examples of distorted use are an expected part of a learning curve. However, before accepting this explanation, I think it is important to honestly consider the following question: Did we anticipate that the large majority of papers achieving PRBs would have undisclosed deviations from their pre-registration plan, or that a top journal would push a PRB on a paper that chose not to pre-register its key analyses? Part of this article’s point is that we should have. In a PRM world, distorted use of pre-registration is exactly what our history and Campbell’s Law would lead us to predict (Klonsky, 2018).

Importantly, the evidence summarized here highlights distorted uses of pre-registration that can be monitored. However, many other kinds of distorted use under PRMs would be nearly impossible to monitor or detect (e.g., see section titled PRARKing). Thus, the available evidence likely *underestimates* the frequency and variety of problems associated with papers motivated by PRM rewards.

Understandably, some may desire more evidence before accepting that PRMs can distort rather than safeguard scientific practice. Unfortunately, because PRMs are relatively new, comprehensive evidence about their impact is not yet available. My hope is that the (a) historical precedents, (b) relevance of Campbell’s Law, and (c) initial empirical evidence described earlier, in combination with the (d) arguments I present in the following sections, are together enough for our field to more critically consider PRMs and alternative ways to fix the replication crisis. Note that many decades before the impacts of misused *p*-values could be fully documented, the use of *p*-values by psychological scientists was referred to as “excessive,” “feeble,” and “pseudorigor” (Meehl, 1978). Perhaps this time, we can anticipate the future and course-correct before years of distorted science accumulates as evidence.

In what follows, I will attempt to imagine the future assuming PRMs continue to position pre-registration as the new indicator for “strong science.” Specifically, I describe how PRMs discourage optimal scientific practices, worsen file drawer effects, create a new analogue to HARKing, and encourage false confidence in studies using pre-registration as a design feature

### PRMs Will Discourage Optimal Scientific Practice

It is critical that we work as a field to encourage optimal scientific practices. However, PRMs are not aligned with this goal. PRMs were designed largely as a straightjacket against a particular type of poor science (i.e., undisclosed researcher degrees of freedom), which is not the same thing as centering and encouraging optimal science.

Consider the following hypothetical. We have a dataset and a simple research question: How do variables A and B relate to each other? When addressing this and any other research question, there will always be multiple reasonable ways to analyze and fully understand the data (Gelman & Loken, 2013). In this example, we might want to (a) compute a Pearson correlation between A and B, (b) compute a Spearman correlation to help rule out an impact from potential outliers, (c) compute correlations between A and B controlling for a potential confounder, (d) compute correlations between A and B controlling for another potential confounder (or several in combination), (e) transform one or both variables before computing the correlation if concerned that non-normal distributions may artifactually impact results, (f) limit analyses to participants who meet reasonable statistical or conceptual inclusion criteria, (g) examine how analyses differ depending on which measure or which combination of measures of A or B are used, and (h) I imagine many others. All these analyses are genuinely useful for understanding and characterizing the relationship of interest.

This consideration also applies to other analytic contexts, such as classification and assessment. Assume we want to evaluate a five-factor structure for a given item-set. Within the framework of confirmatory factor analysis, we might want to (a) use maximum likelihood estimation and associated fit indices, (b) use diagonally weighted least squares given that items are on non-continuous true-false or Likert-type scales (even though fit indices will be more difficult to interpret), (c) consider models in which hypothesized factors are and are not allowed to correlate, (d) consider models in which hypothesized factors do and do not indicate possible higher-order factors, (e) consider additional models suggested by (unexpected) findings of initial analyses, (f) consider additional models suggested by the literature, and (f) many other possibilities. Again, all these analyses are genuinely useful for understanding and characterizing the structure of the item-set. To omit one is to omit relevant information.

Clearly, if someone conducts numerous relevant analyses with the aims of selectively reporting the most “publishable” ones, or the ones that best fit a particular perspective, that would be poor science and somewhere between negligent and fraudulent. Perhaps mandatory pre-registration is a useful straightjacket for such researchers. But how should the ideal scientist, who wants to achieve and convey a full understanding of their data, approach this situation?

I would argue that the ideal scientist should be interested in conducting all these analyses and would only feel they have achieved maximum understanding after conducting all the relevant analyses they can think of. The multiverse of analyses will yield confidence in the robustness of findings if they converge on a particular pattern, and they will suggest nuance, caution, and potential fragility if they do not. They are all useful and relevant, and with rare exceptions, to prioritize one for having been pre-registered is arbitrary. Consequently, a “multiverse” approach to data analysis—an approach that embraces a range of reasonable analytic options—is scientifically optimal for fully understanding effects and patterns of interest (Steegen et al., 2016).

PRMs discourage this ideal in several ways. First, they establish as a norm that researchers should choose one or a subset of reasonable analyses and not others. Consider a situation in which there are eight reasonable ways to analyze a dataset for a given research question about the relationship between two variables. The fullest understanding would be achieved by examining the pattern of findings from all eight. In contrast, PRMs are structured such that researchers who choose and report just one are rewarded. I would suggest this is poor science. What if results for other reasonable analytic approaches offer a different answer, are we to defer to the one that happened to be pre-registered? Why would I not care equally about the results for the other seven reasonable analytic approaches? Why should we award a badge for prioritizing one of the approaches a priori? Why should we award a badge for studies that omit or ignore reasonable analytic approaches beyond the one they pre-registered? PRMs harm scientific practice by offering a blanket reward for a selective rather than comprehensive approach to understanding data, even though the latter approach is optimal.

Second, by rewarding adherence to data analysis plans before the data are engaged with, PRMs devalue analyses conceived after engaging with the data. From the PRM perspective, only the former is badge-worthy, and the latter perhaps worthy of suspicion. Critically, there is no inherent scientific reason why the analyses the researcher thought of first are more informative than the ones the researcher thinks of later (a point highlighted by Pham & Oh, 2021). In fact, analyses conceived after engaging with the data are often *more* important and *more* informed. Consider the following true anecdote.

Early in my career, I analyzed a particular psychophysiological index in relation to a diagnostic questionnaire and found a “significant” relationship. I was excited because such a finding is quite publishable, and I was pre-tenure. Also, although this was roughly 2006 and I had not pre-registered my design, this was the analysis I had been planning for months and absolutely would have been the focus of my pre-registered plan. Had I pre-registered the analysis, I could have proceeded to the write-up, knowing not only that my chances of publication were strong given the finding but also that I had done “strong” science and would be recognized by a PRB. However, there were no PRMs or PRBs, and I very much wanted to feel confident about anything I published. It occurred to me that the psychophysiological measure had potential outliers that could impact results. I found a clear outlier, re-ran the analysis without it, and the result disappeared. I also ran the analysis using a second validated (though less comprehensive) measure of the diagnostic construct in the dataset and did not find the relationship. As a result I did not submit a false-positive finding for publication.^1^ However, in a PRM world, this type of situation is likely to have a different ending: not only a published false-positive finding, but one accompanied by a PRB vouching for its credibility.

While the aforementioned information is only an anecdote, it illustrates the importance of robustness checks (Nuijten, 2022; Steegen et al., 2016). We can only evaluate the robustness or fragility of particular statistical findings by examining what happens when different reasonable versions of the analysis are conducted. Converging findings support robustness, and diverging findings suggest fragility. Many ideas for robustness checks will only be stimulated after engaging with the data. This is not a problem scientifically! Ideal data analytic practice requires constant care and thought both before and after engaging with data. However, by selectively rewarding only one part of the data analytic enterprise, PRMs deincentivize other critical parts of the process, including robustness checks, and even imply that post hoc analyses are of inferior relevance when the opposite is often true.

In other words, PRM incentives such as PRBs can be achieved and will convey that findings are extra trustworthy, even when no robustness checks have been conducted—and even if other reasonable analytic approaches yielding divergent results have been ignored or omitted. This is harmful. It hurts science to award badges suggesting that fragile findings are trusted findings because a particular design feature has been used.

Third, PRBs discourage thoughtful science by encouraging checklist science. Checking certain boxes enables researchers to earn badges, which then become proxies for scientific strength. However, we have already been down that road. We used to give “checkmarks” for other tools of science that became proxies for scientific strength, like hypotheses, *p*-values below .05, and multi-study designs. The result is that researchers prioritized the checkmarks, the cosmetic presence of the indicators of “strong science,” more than thoughtful, nuanced, and careful scientific practice. Replacing old checklist items with new checklist items perpetuates this historical pattern.

I can anticipate a rebuttal: that pre-registration can accommodate and even support the scientific ideals I described earlier (e.g., helping to plan multiverse analyses and robustness checks) if done well. Note that the same could be said about *p*-values, hypotheses, and multi-study designs: They are all tools that fully support robust science when done well. The “done well” part is the crux. Per Campbell’s Law, morphing these tools (pre-registration, *p*-values, hypotheses, multi-study designs) into indicators and goals creates conditions that ensure they are used the opposite of well.^2^ We must learn that when we canonize a tool, it becomes a goal, and its use and impact on science becomes distorted, not optimized. In contrast, the use and impact of pre-registration will be optimized if it remains one of many useful tools to be used thoughtfully and purposefully.

### File-Drawer 2.0

PRMs will also create a new, arguably more harmful, version of the file drawer problem. One reason why false-positive findings are disproportionately published has been termed the “file drawer” effect (Rosenthal, 1979). In short, because journals prefer to publish “significant” findings (e.g., *p*-values below .05), larger effects are more likely to be published in journals, whereas small or negligible effects are more likely to end up in “file drawers.” As a tool, pre-registration has been well-positioned to reveal these patterns. For example, if there is suspicion that published findings for an effect of interest may be impacted by this bias, a high-powered pre-registered design can yield a more precise and less-biased estimate of the effect.

However, the conversion of pre-registration from a tool to a badge/goal will cause a new and potentially more harmful version of the file drawer problem. The aim of PRMs is to make pre-registration “the norm” (Nosek et al., 2018). In other words, scientists are being encouraged to pre-register, and to view non-pre-registered work with suspicion, without thinking too hard about the relevance of pre-registration to the particular research question and dataset and without carefully considering the strengths and weaknesses of pre-registration relative to other design features that can support robust science. This may sound like a good idea, but remember what happened when scientists were encouraged to make hypotheses, *p*-values below .05, and multi-study designs the norm without thinking too hard about their use: There are surprising consequences.

Consider a future in which labs make pre-registration the norm. Note that this new reality will not affect the fundamentals of the file drawer problem. As always, these labs will be conducting numerous studies at any given time, only some of which will ultimately be published. Also as always, the factors that influence which studies are written up and submitted for publication and which of these studies are chosen by journals for publication include whether the hypothesis was confirmed or whether interesting effects were obtained (Schmucker et al., 2014). In other words, the file drawer problem persists as usual but with a key difference: The published findings are now accompanied by PRBs vouching for their scientific credibility. Depending on your perspective, this is either the same file drawer problem as before or worse given that it ensures the usual false-positive findings are now accompanied by badges attesting to their trustworthiness.

A key point is the discrepancy in the impact of pre-registration depending on whether it is a tool or a goal. As a tool, pre-registration can help remedy the file drawer problem: When we suspect publication bias has inflated the magnitude of an effect of interest, pre-registration can put that effect to the test in a careful, high-powered, unbiased design. In contrast, as a goal and norm for publication, PRM rewards like badges create false confidence in studies that are just as susceptible to publication bias and file drawer effects as they always have been.^3^

### PRARKing

After hypotheses became a goal rather than a tool, scientists changed how they thought about and used hypotheses and learned to HARK. HARKing took many forms, some of which were considered deceptive, some of which became socially normative and encouraged (Bem, 2003), and many of which fell somewhere in the murky in-between (Kerr, 1998). It is important we recognize that the extent and variety of ways to HARK were not outliers of human behavior but expectable and inevitable per Campbell’s Law once hypotheses became a goal. Similarly, if pre-registration becomes a goal rather than a tool, PRARKing (pre-registering after results are known; Yamada, 2018) will inevitably occur to an extent and variety we cannot fully imagine or prevent.

Some versions of PRARKing will be fraud. For example, scientists will learn that papers meeting criteria for PRBs are given better reviews and more credence than articles without, and dishonestly pre-registered analysis plans after results are known. This fraud will be easy to conceal. Notably, while PRARKing is likely if pre-registration is a badge and a goal in and of itself, it would be far less likely if pre-registration were viewed as one of many useful tools of science. In short, PRMs will unnecessarily incentivize fraud that will be difficult to detect.

I suspect other forms of PRARKing will be more common. Much like versions of HARKing became acceptable and even encouraged (Bem, 2003), PRMs make it likely that subtle versions of PRARKing will emerge and become common. Consider this hypothetical correspondence between a grad student and a PI who value participation in the open-science and PRM movement:

Student: Professor X, I just noticed a really cool relationship between variables A and B in the BLANK dataset!

Professor: That’s exciting! Stop what you’re doing, write up the pre-reg, and then we’ll dig in!

If this potential scenario sounds far-fetched, I would suggest one is not paying sufficient attention to human nature and our field’s history.

In sum, as a tool, pre-registration is a useful and sometimes critical feature for many study designs. However, as a mandate, pre-registration will cause a wide range of PRARKing behaviors that will come in many flavors and be difficult or impossible to detect. The result will be poor and sometimes fraudulent science hidden behind a badge vouching for its credibility—much like poor science used to hide behind the veneer of hypothesis-driven work, *p*-values with asterisks, and multi-study designs.

### False Trust in Fragile Findings

An overarching problem with PRM policies such as badges is that they will generate false confidence in studies that achieve them. This will happen in several ways, including some already mentioned. For example, I have already noted that PRBs and other PRM rewards are routinely given to publications (a) with undisclosed deviations from their pre-registered plan, (b) without robustness checks for the fragility of findings, (c) with the usual susceptibility to file-drawer effects, and (d) that involve various forms of PRARKing. However, PRMs will create other forms of false confidence as well.

Anecdotally, I have heard colleagues say and seen colleagues tweet things like “you can trust the findings because the study was pre-registered” and “badges are a quick and easy sign that I can trust this study more.” These kinds of statements suggest a distorted understanding of the factors that impact the reliability of a study’s findings. Even in optimal circumstances—perfectly faithful and honest implementation of a pre-registered plan—pre-registration only inoculates against misuse of researcher degrees of freedom, but does not inoculate against the normal sources of sampling error and normal forms of measurement error that can lead to inaccurate effect-size estimates. Thus, pre-registered or not, any given study result on its own cannot be “trusted.” Period. All effects require replication before they can be trusted, and can only be trusted to the extent they replicate in sufficiently large samples and across multiple measures, recruitment procedures, contexts, and investigative teams. This remains true regardless of whether or not the study was pre-registered!

In short, regardless of pre-registration, replication is the only arbiter of replicability. I would suggest that PRMs distract from this essential fact. Instead, PRM mechanisms such as badges create false confidence in the robustness of any given study’s findings and downplay the universal need for replication.

## We Must Suppress Our Affinity for Aesthetic Markers of Strong Science

In a 1974 commencement speech, renowned physicist and Nobel Prize winner Richard Feynman warned against efforts that mimic the precepts and forms of scientific methodology while lacking essential qualities of genuine scientific inquiry.^4^ Psychological science has a long history of unwittingly engaging in this practice. For decades, hypotheses, *p*-values below .05, and multi-study designs served as aesthetic features that gave the look and feel of strong, trustworthy science—even when the substance and robustness of findings were lacking. The aesthetic value of these features drove decisions by researchers and journal editors about how and what to publish, and the original purposes of these tools of science became lost. The result was publication of an enormous number of papers with non-replicable findings.

PRMs appear to be the new aesthetic (or “performative”; McDermott, 2023) marker for desirable science. They convert pre-registration from a useful tool into a precept or form—a veneer, often a literal badge—of strong science. History illustrates what will happen: PRMs will distort scientific practice and harm our field. Initial evidence suggests this is already happening: There is a large gap between the appealing veneer of PRM badges and the substance of studies they are awarded to (Claesen et al., 2021; van den Akker et al., 2023). Because PRMs are touted as safeguarding scientific robustness and integrity, they are possibly even more dangerous than the previous generation of aesthetic markers that unwittingly harmed scientific robustness and integrity. Per Feynman’s warning, we must cease our affinity for aesthetic markers of strong science if we are to avoid a repetition of history and safeguard the future of psychological science.

## An Alternative Vision for Improving Psychological Science

If the path to robust science is not selecting certain tools to canonize, what is it? I believe the answer may sound unattainable, but in actuality, it is eminently achievable: We must create a culture that reveres and celebrates robust findings as much as it once revered and celebrated flashy but fragile findings. In this new context, sociocultural forces are the primary straitjacket against abusing researcher degrees of freedom, and PRMs have little benefit with high risk of harm. In this new context, scientists will prioritize study design features that increase the likelihood of producing replicable findings just like they used to prioritize features that produced flashy but fragile findings.

This vision begs the following question: Which study design features should scientists choose to support replicability? There are many. Some basic examples include large sample sizes (Szucs & Ioannidis, 2017), valid measurement (Lilienfeld & Strother, 2020), and robustness checks for key analyses (Steegen et al., 2016). However, we must not create checklists or badges for these (or other) features, as there are always exceptions, for example, single case-experimental designs and pilot/feasibility studies (sample size exception), phenomena requiring provisional or novel assessment when validated measures are lacking (measurement exception), and replication studies targeting specific analytic approaches used in past studies (robustness checks exception). We must also understand that confidence in the replicability of findings from a single study should usually be low (regardless of whether the study was pre-registered or published with badges) and that confidence in findings only increases to the extent that they were derived using valid measures, in large samples, with checks for robustness across reasonable analytic approaches, and that they ultimately replicate across samples and investigative teams.

Not only is this vision of sociocultural change and reinforcement achievable, but it is happening. In fact, if it were not for PRMs, I would be excited to write that the massive changes happening in the field are exactly the kinds needed to create this new culture. These include a substantial increase in the number and variety of efforts to investigate replicability and uncover fragile findings (e.g., APS, 2023b; OSC, 2015; datacolada.org; replicats.research.unimelb.edu.au); opportunities to publish studies regardless of whether they yield “significant” results (Chambers & Tzavella, 2022; Scheel et al., 2021); editorial board decisions that put emphasis back on methods and results by rolling back word limits for these sections (APS, 2023a); the founding of influential organizations that champion robust science such as the Society for the Improvement of Psychological Science (https://improvingpsych.org) and the Center for Open Science (https://www.cos.io); and the proliferation of papers highlighting features that help support replicability, including large sample sizes (Szucs & Ioannidis, 2017), valid measurement (Lilienfeld & Strother, 2020), analytic robustness checks (Steegen et al., 2016), and replication across samples and investigative teams (Allen et al., 2023; OSC, 2015; Simons, 2014).

A culture that values replicability is what most reinforces the pursuit of robust, replicable science—not badges or checklists. If you are skeptical, consider that researchers spent decades producing flashy, fragile science not because they were given badges or other manufactured incentives, but because of the sociocultural processes and institutions that rewarded flashy, fragile science. These processes and institutions are now changing. All of us scientists are learning that if we publish fragile findings, we will be found out. We are also learning that if we produce robust, replicable science, we will be valued—by journals, by our colleagues, by our field.

PRMs are positioned as a way to reinforce strong science, but in reality, they reinforce achieving badges. In contrast, genuine culture change creates the desired context of reinforcement and punishment. To quote Richard Feynman, who was speaking about his own field of physics:

We’ve learned from experience that the truth will come out. Other experimenters will repeat your experiment and find out whether you were wrong or right. Nature’s phenomena will agree or they’ll disagree with your theory. And, although you may gain some temporary fame and excitement, you will not gain a good reputation as a scientist if you haven’t tried to be very careful in this kind of work (Feynman, 1974).

In psychological science, we are now doing the work we need to do to create a culture of robust, replicable science. It is time for PRMs and other checklist, aesthetic, and performative solutions to be left behind.

## Epilogue

To echo the words of Boss Tanaka from the movie *Kill Bill*, I write this article in service of psychological science, a field “I love.” I cared deeply during those many years when false-positive and fragile results were routinely published and lauded, and I care deeply now as the field embraces new policies, some of which in my mind continue harmful trends of the past. I believe I share the same goal as those advocating PRMs: a field that produces, rewards, and reveres replicable, robust science.

After sharing his thoughts, Boss Tanaka was swiftly decapitated in front of his peers. I hope I have done better.

## Notes

1. Obviously it would have been better to pursue publication of the findings regardless. Shortly thereafter, I became more skilled at publishing negligible effects for key analyses (e.g., Glenn et al., 2011; Klonsky & May, 2010).

2. While this article focuses on how PRMs can harm scientific integrity, others make even broader arguments that PRMs suppress scientific creativity and disadvantage junior scholars (McDermott, 2023).

3. This kind of publication bias could potentially be mitigated by the registered report format (Chambers & Tzavella, 2022) or by reviews/meta-analyses of archived pre-registration plans; however, in either case, PRM mandates would remain vulnerable to the overarching concern raised in this article (i.e., morphing a tool into a goal), as well as the other specific concerns raised about PRMs (i.e., discouraging optimal scientific practice, encouraging PRARKing, and generating false confidence in fragile findings).

4. Feynman famously referred to this tendency as “cargo cult science,” but this term has colonial and prejudiced origins so I have opted not to center it.
